# Surgical site infection and costs in low- and middle-income countries: A systematic review of the economic burden

**DOI:** 10.1371/journal.pone.0232960

**Published:** 2020-06-04

**Authors:** Mark Monahan, Susan Jowett, Thomas Pinkney, Peter Brocklehurst, Dion G. Morton, Zainab Abdali, Tracy E. Roberts

**Affiliations:** 1 NIHR Global Health and Global Surgery Unit, Institute of Translational Medicine, Heritage Building, University of Birmingham, Birmingham, England, United Kingdom; 2 Health Economics Unit, Institute of Applied Health, College of Medical and Dental Sciences, University of Birmingham, Birmingham, England, United Kingdom; 3 Birmingham Surgical Trials Consortium, Institute of Applied Health, College of Medical and Dental Sciences, University of Birmingham, Birmingham, England, United Kingdom; University Magna Graecia of Catanzaro, ITALY

## Abstract

**Background:**

Surgical site infection (SSI) is a worldwide problem which has morbidity, mortality and financial consequences. The incidence rate of SSI is high in Low- and Middle-Income countries (LMICs) compared to high income countries, and the costly surgical complication can raise the potential risk of financial catastrophe.

**Objective:**

The aim of the study is to critically appraise studies on the cost of SSI in a range of LMIC studies and compare these estimates with a reference standard of high income European studies who have explored similar SSI costs.

**Methods:**

A systematic review was undertaken using searches of two electronic databases, EMBASE and MEDLINE In-Process & Other Non-Indexed Citations, up to February 2019. Study characteristics, comparator group, methods and results were extracted by using a standard template.

**Results:**

Studies from 15 LMIC and 16 European countries were identified and reviewed in full. The additional cost of SSI range (presented in 2017 international dollars) was similar in the LMIC ($174—$29,610) and European countries ($21—$34,000). Huge study design heterogeneity was encountered across the two settings.

**Discussion:**

SSIs were revealed to have a significant cost burden in both LMICs and High Income Countries in Europe. The magnitude of the costs depends on the SSI definition used, severity of SSI, patient population, choice of comparator, hospital setting, and cost items included. Differences in study design affected the comparability across studies. There is need for multicentre studies with standardized data collection methods to capture relevant costs and consequences of the infection across income settings.

## Introduction

Mortality within 30 days of surgery is the third largest contributor to global deaths [1]. Surgical Site Infection (SSI) is linked to 38% of deaths in patients with SSI [2]. SSI is common, associated with increased patient morbidity and mortality [3, 4], recognised globally as a problem and shown to represent a substantial financial burden [5, 6]. In comparison to the relatively high income countries (HIC) of Western Europe, the incidence rate of SSIs is much greater in Low- and Middle-Income Countries (LMIC) [7, 8] and here the majority of the hospital care cost is borne by the patient [9]. In the LMIC setting, the risk of acquiring an SSI substantially increases the overall risk of financial catastrophe- a situation in which health care spending on this event exceeds 10% of annual household expenditure [10].

Identifying appropriate solutions to combat SSI is of global interest [6, 11, 12]. Recently completed and ongoing research studies to find the most cost-effective prevention strategies for SSI, are having mixed success [13, 14]. The majority of this research is randomised controlled trials (RCTs) with a parallel economic evaluation based in HIC [15, 16]. Plans are in place to carry out similar studies exploring cost-effective strategies to combat SSI in the LMIC setting [17]. Significant challenges hamper clinical trials in LMICs relating to lack of infrastructure and limited human resources [18]. This limits the data that can be feasibly collected in contrast to trials in HICs settings.

A cost of illness (COI) study quantifies how much society is spending on a particular disease and represents the cost burden averted if the disease was eradicated [19]. Understanding the additional cost burden imposed by the complications of surgery such as those caused by an SSI, helps to strengthen the case for identifying interventions to reduce such complications [20]. This in turn provides the justification for undertaking economic evaluations to present relevant evidence to inform the prioritization of resource allocation decisions for interventions to reduce SSI complications.”.

We identified five main challenges in measuring the additional costs associated with an SSI. First, different definitions of an SSI affects which patients are considered to have an SSI [21]. Second, as an SSI can manifest beyond hospital discharge, approaches for post-discharge SSI confirmation will impact SSI detection rate [22, 23]. Follow-up difficulties can be exacerbated for surgical patients in low income settings due to high out-of-pocket transportation costs in accessing healthcare [24].

Third, estimating the additional cost of SSI relies on the choice of the comparator, which is patients without SSI. Studies with a case-control design try to address potential confounding with an adjusted comparison where each of the exposure and control patients have matching confounding variables (e.g. same age, gender, surgical procedure). Yet, the choice of matching variables should be considered carefully in case-control studies because of its impact on the efficiency and validity of the results [25].

Fourth, SSI costs are only as representative as the hospital settings used. Resource use and costs are known to differ across urban and rural settings and different patient population mixes from different surgical procedures can influence the cost of SSI, limiting the generalisability across procedures. Finally, SSIs vary in severity, and those SSIs that are severe can substantially increase costs and inpatient length of stay [26]. However, the distinction between SSI severity levels is open to subjective interpretation by the attending physician [27].

The objective of this study is to critically appraise and assess how the cost of SSI has been estimated in a range of LMIC studies and compare with a selection of high income European studies which explored similar SSI costs. European studies are included in the review to provide a reference standard for the LMIC studies. The aim of the comparison is to examine the costs associated with SSI (presented in international dollars) across the different settings and identify potential data gaps, and methodological considerations in each setting.

This paper is structured so that the review of the selection of European studies is presented in Part 1. An analogous review of the LMIC studies is presented in Part 2. Part 3 presents a comparison between the main finding of the reviews for the HIC and LMIC settings before the main discussion.

## Materials and methods

The review followed the UK Centre for Review and Dissemination [28] guidelines and Preferred Reporting Items for Systematic Reviews and Meta-Analyses (PRISMA) [29].

### Search strategy

The following electronic databases were searched from inception to 20^th^ February 2019: EMBASE and MEDLINE In-Process & Other Non-Indexed Citations. Additional references were found using hand searching of relevant journal articles and Google scholar searches. Search terms used for each database are detailed in S1–S4 Files

### Eligibility criteria

Studies were included if they considered the costs associated with SSIs in European Organisation for Economic Co-operation and Development (OECD) countries [30]. For the analogous review of LMIC, studies were included if they considered the economic impact of SSIs in LMICs. For both settings costs could be borne by the healthcare providers, patients, wider community and/or society. Eligible articles included cost analysis, partial or full economic evaluations (trial-based and model-based) and cost of illness studies in a European country or LMIC setting. Multi-country studies were included if at least one eligible country was included and the study’s findings were reported separately for that country. Non-eligible studies were those that were not published in English, conference proceedings, protocols, commentaries, and editorials.

#### Study selection

The titles and abstracts of the databases’ search results were screened against the eligibility criteria. A three stage categorisation process was used to determine relevant studies appropriate for inclusion, using methods described elsewhere [31]. Two investigators carried out study screening and data extraction for the LMIC search (MM & ZA). One investigator (MM) carried out all study screening and data extraction for the European literature search, and another investigator (ZA) undertook screening of a random 20% to assess agreement. Disagreements were resolved through discussion, a third independent investigator (TR) was sought where agreement could not be reached.

For each included study, data were extracted on the study characteristics, country setting, costs and resource use included, use of adjusted analyses, and the main results reported. The information was tabulated, and the issues faced by the individual studies in estimating the additional costs of SSI were compared narratively. For consistency across studies, costs were converted to international dollars and inflated to 2017, where appropriate. To improve comparability of cost findings, costs were adjusted by their country’s Purchasing Power Parity (PPP) conversion factor [32]. Where a country did not have a PPP conversion factor, an implied PPP conversion factor from the IMF was used instead [33]. For inflation purposes, studies without a specified cost year were assumed to be the last year of data collection.

All included studies were assessed by a modified reporting Müller checklist (translated into English) for COI studies and scored by their inclusion of relevant items [34]. A study scored one on each aspect they had described or justified out of a possible maximum score of 36. The checklist for each study is available upon request.

## Results and discussion

### Part 1: European literature search

**Fig 1:**
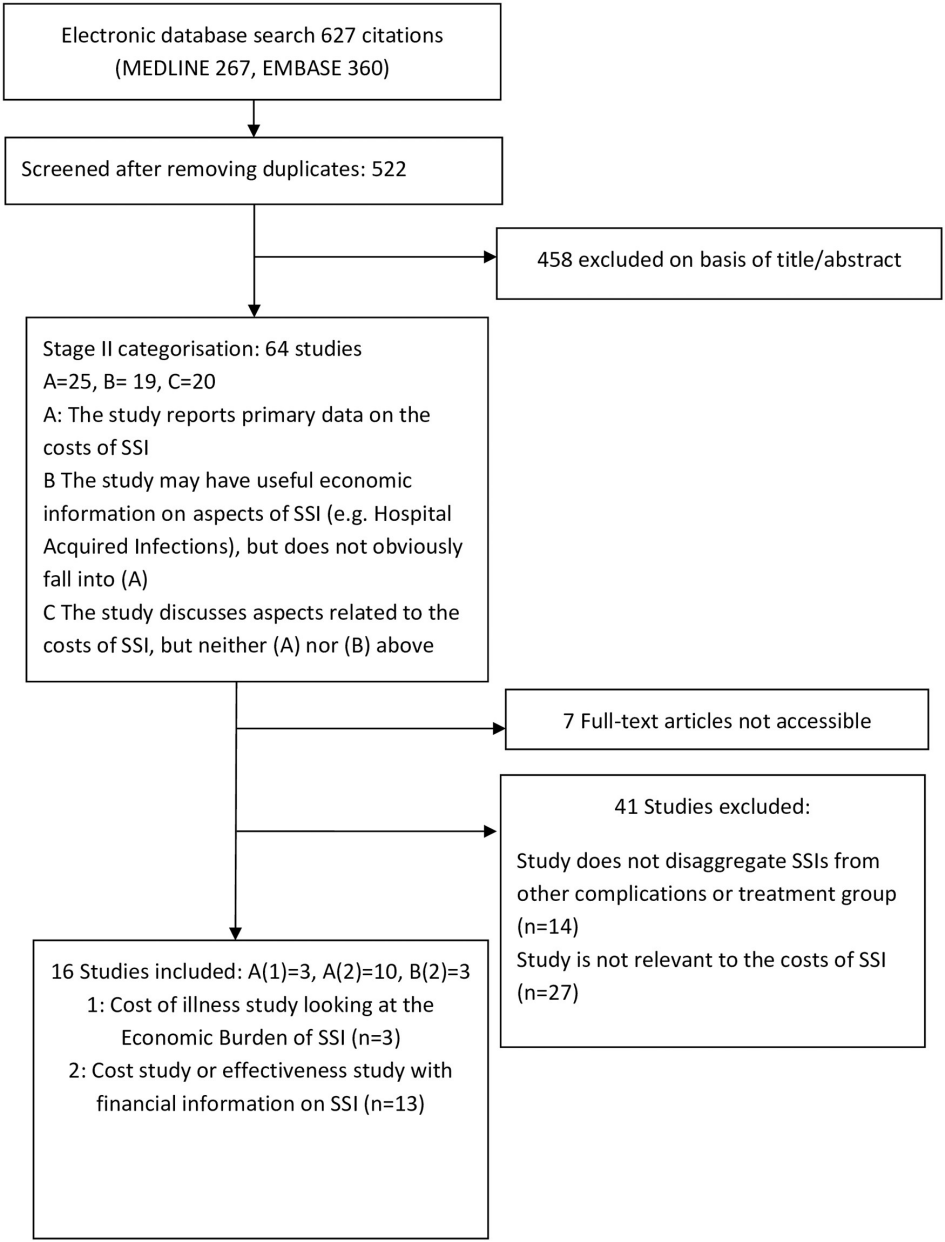
PRISMA diagram of European search.

The electronic database search for the European studies yielded 588 citations. Fig 1 presents a flow diagram of the selection process. Sixteen studies met the inclusion criteria.

#### General study characteristics

The sixteen studies were published from 1992 to 2018 and data collection spanned 1987 to 2016. Studies were based in England (n = 6) [35–40], Spain (n = 2) [41, 42], Scotland (n = 2) [43, 44], Finland (n = 1) [45], France (n = 1) [46], Switzerland (n = 1) [47], Belgium (n = 1) [48], Denmark (n = 1) [49] and Germany (n = 1) [50]. Table 1 shows general characteristics of each study included in the review.

**Table 1:** European study characteristics.

Lead author (Year)	Country	Patient population	Setting	Study aim	Type of study	Number of SSI & Comparator	Period of data collection
**Cardiothoractic surgery**							
Graf (2010) [50]	Germany	CABG patients	University hospital	Calculate the costs of deep sternal wound infection	Case-control study	17 SSI/ 34 Non-SSI	2006–2008
**Colorectal surgery**							
Tanner (2009) [37]	England	Adult colorectal patients	University hospital	Provide an accurate cost for treating patients with SSI	Surveillance study	29 SSI/ 76 Non-SSI	2008
Turtiainen (2010) [45]	Finland	Vascular surgery patients	Four secondary referral hospitals	Calculate the extra cost of services needed to treat SSI	Prospective observational study	49 SSI /136 Non-SSI	2007–2008
**Multiple surgical categories**							
Alfonso (2007) [41]	Spain	Adult patients	General, tertiary hospital	To identify overall costs generated by SSI patients	Cost of illness study	30 SSI/ 52 non-SSI	2001–2005
Defez (2008) [46]	France	Acute care patients	University hospital	Calculate the additional costs of nosocomial infection:	Prospective cohort study	21 SSI/21 non-SSI	2001–2003
Jenks (2014) [35]	England	Patients who underwent major surgical procedures	University hospital	Determine the clinical and economic burden of SSI	Cost analysis	282 SSI/ 14,018 non-SSI	2010–2012
Lynch (1992) [43]	Scotland	Adult surgical patients	Teaching hospital	Study the cost of SSI	Cost analysis	513 SSI/ 2969 non-SSI	1987–1989
Reilly (2001) [44]	Scotland	Surgery patients	Unspecified hospital	Quantify the cost of SSI to the hospital, community, and patient	Prospective cohort study	220 SSI /1982 non-SSI	1995–1999
Vegas (1993) [42]	Spain	General surgery and digestive surgery patients	University hospital	Estimate the length of stay of SSI patients	Prospective cohort study	106 SSI/ 212 non-SSI	1990
Vrijens (2012) [48]	Belgium	Acute care patients	Acute care hospitals in Belgium	Estimate the total economic cost of infection to the public healthcare provider	Retrospective cohort study	77 SSI/ 261 non-SSI	2007
Weber (2008) [47]	Switzerland	Traumatology, visceral and vascular surgery patients	University hospital	Quantify the economic burden of SSI	Retrospective cohort study	168 SSI/ 168 non-SSI	2000–2001
**Gynaecological surgery**							
Hyldig (2018) [49]	Denmark	Obese women after caesarean section	5 obstetric departments across 2 tertiary & 3 teaching hospitals	Evaluate the cost-effectiveness of incisional negative pressure wound therapy in preventing SSI	Within trial cost effectiveness analysis	57 SSI/780 non-SSI	2013–2016
**Orthopaedic surgery**							
Edwards (2008) [40]	England	Hip fracture patients	University hospital	Estimate the cost of treating SSI	Retrospective cohort study	80 SSI/ 80 non-SSI	1999–2004
Pollard (2006) [36]	England	Proximal femoral fracture surgery patients over 65 years	Tertiary teaching hospital	Assess the financial burden of deep SSI after surgery	Retrospective cohort study	61 SSI/ 122 non-SSI	1998–2003
Parker (2018) [38]	England	Lower limb open fracture patients	24 specialist trauma hospitals	Estimate economic outcomes associated with deep SSI	Costing analysis of a prospective RCT	35 SSI/ 423 non-SSI	2012–2015
Thakar (2010) [39]	England	Proximal femoral fracture patients	Tertiary teaching hospital	Calculate the additional hospital costs due to complications	Prospective cohort study	46 SSI/ 92 non-SSI	2003–2008

All costs were inflated and converted to 2017 international dollars where appropriate

NNIS, Nosocomial Infection Surveillance System risk index; SSI, Surgical Site Infection;

#### Definition of SSI

SSI was defined using the Center of Disease Control (CDC) guidelines in most of the studies [35, 37, 38, 41, 42, 45, 46, 50]. Other strategies for SSI confirmation included using a microbiological test [36, 39] or if a patient required antibiotic treatment for wound problems [40, 49]. Lynch et al [43] defined an SSI based on pus discharge or a wound with a score of greater than ten on ASEPSIS, a scoring mechanism for postoperative SSI [51]. Reilly et al [44] defined an SSI as pus or painful skin inflammation indicative of cellulitis.

Patients were followed-up for the occurrence of SSI for at least 30 days [35, 41, 43, 44] with two studies following up SSI patients until the wound had healed [37, 45]. Approaches to diagnose post-discharge SSI included outpatient clinics or primary care visits [41, 44, 45], surveys/questionnaires, [35, 43] or a home visit [37].

#### Patient matching

An imbalance of patient characteristics can bias and confound the cost calculation of SSI patients. This is analogous to an observational non-RCT setting where the difference in outcomes may be partially or wholly explained by factors other than the presence of SSI. Some form of patient matching in the analysis to adjust for confounding variables was used in most studies [35, 36, 38, 39, 41, 42, 46–48, 50]. However, justification for the selected matching variables was given in less than half of these studies [36, 38, 39, 41, 48].

#### Setting & procedure

Public teaching hospitals [35–37, 39–43, 46, 47, 50] were the setting for majority of the studies with one hospital setting unclear [44], and another study referring to unspecified referral hospitals [45]. Study settings were mostly restricted to a single site with only four studies involving multiple hospitals [38, 45, 48, 49]. Surgical procedures ranged from general surgery or multiple surgery categories (n = 8), cardiothoracic (n = 1), colorectal (n = 2), gynaecological (n = 1), and orthopaedic (n = 4). The patient population were all adult patients.

Half the studies that assessed SSI across surgical categories reported surgery category-specific costs associated with SSI [35, 43, 44, 47]. All of these studies showed variation of SSI costs across surgical categories. Severity of SSI was always associated with increased costs. A deep SSI was more costly compared to a superficial SSI in all studies that had severity-specific SSI costs [39, 47, 49]. Yet, the stated approaches to classify the superficial versus deep SSI differed. Approaches to define superficial SSI included CDC criteria [49], or a treatment for an infection at the surgical site within 30 days postoperatively [47], or were not defined [39]. Approaches to classify a deep SSI included a microbiological confirmation of tissue from a further surgery [39], or an SSI requiring surgery [47] or using CDC criteria [49] The sample size of SSI patients in the European studies ranged from as low as 17 patients to as high as 513 patients.

#### Cost components

The type of costs included and considered in each of the studies is shown in Table 2 and S1 Table. All studies considered at least some form of direct medical costs in their cost calculations. However, there was a considerable variation in the description and the number of direct medical cost items included in each study. In terms of the costs arising from the initial hospitalization of patients, the description of the included cost components ranged from an unspecified cost per bed day to a comprehensive bottom up costing of the hospital length of stay, consumables, diagnostics, overhead, reoperation and staffing costs. Non-hospital costs were also considered in some of the studies including post-discharge costs from general practitioner/ nurse visits [37, 41, 43, 44], and patient/community costs of wound dressings.

**Table 2:** Costs of SSI in European studies.

Lead author (Year)	Adjusted group comparison	Costs included	Average cost SSI patients	Average costs Non-SSI patients	Additional cost of SSI	Length of Stay
**Cardiothoractic surgery**						
Graf (2010) [50]	Age, sex, DRG, preoperative LOS	Surgery, lab tests, hospital LOS	$50,912	$18,751	$32,161	SSI: 34.4 days
						Non-SSI: 16.5 days
**Colorectal surgery**						
Tanner (2009) [37]	Unadjusted analysis	Hospital stay, nurse & GP visits, outpatient clinic, wound dressing, readmissions, antibiotics, wound swab	Not reported	Not reported	$18,101	SSI: Extra 22.72 days
						Non-SSI not reported
Turtiainen (2010) [45]	Unadjusted analysis	LOS, Outpatient clinic and rehabilitation	Not reported	Not reported	$4,237	Not reported
**Multiple surgical categories**						
Alfonso (2007) [41]	Age, sex, diagnosis, surgery duration, comorbidity, and procedure	Hospital Stay, readmission, diagnostics, antibiotics informal care, primary care, productivity loss	Not reported	Not reported	Health care costs: $15,263	SSI pre-discharge: 23.73 days
					Informal care: $15,734	SSI post-discharge: 12.99 days
					Societal costs: $145,336	No SSI: 9.45 days
Defez (2008) [46]	Age, sex, ward type, principal diagnosis	Hospital stay, laboratory tests, radiology, surgery, diagnostics, & antibiotics	Not reported	Not reported	$2,780	Not reported
Jenks (2014) [35]	Surgery, age and NNIS risk index	Overhead, staffing costs, readmission, reoperation, hospital stay, diagnostics, consumables	$12,928	$5,837	$5,239	SSI: 19 days
						Non-SSI: 5 days
Lynch (1992) [43]	Unadjusted comparison	GP visits, wound dressings, antibiotic costs, hospital stay	$3,678	$2,116	$1,563	No overall figures reported
Reilly (2001) [44]	Unadjusted comparison	Hospital stay, readmissions, GP and nurse visits, wound dressings, antibiotic prescriptions	Not reported	Not reported	$541	Not reported
Vegas (1993) [42]	Diagnosis, procedure, age	Hospital stay	Not reported	Not reported	$10,688	SSI: extra 14.33 days
Vrijens (2012) [48]	Destination after discharge, hospital, comorbidity, ward, Age, DRG	Hospital stay	Not reported	Not reported	$3,149	SSI: 35.2 days
						Non-SSI: 29.2 days
Weber (2008) [47]	Age, procedure, and NNIS risk	Antibiotic use, postoperative LOS, hospital costs and patient charges	Not reported	Not reported	Overall: $17,060	SSI: 29 days
					Superficial $2,226	
					Deep incisional: $3,801	
					Organ space: $34,001	Non-SSI: 12.3 days
**Gynaecological surgery**						
Hyldig (2018) [49]	Unadjusted analysis	Inpatient stays, outpatient care, antibiotic treatment, postoperative dressing, primary care visits	Not reported	Not reported	Superficial SSI: $21	Not reported
					Deep SSI: $9,527	
**Orthopaedic surgery**						
Edwards (2008) [40]	Unadjusted analysis	Inpatient stay, equipment, surgery consumables and staff salaries, investigations, medication, antibiotics	$49,290	$17,060	$32,229	SSI: 76 days
						Non-SSI not reported
Parker (2018) [38]	Age, sex, trial site, wound grade, diabetes, height, weight, and smoking status	Hospital inpatient & outpatient services, community health & social care, medication, aids and adaptations	$22,255 (complete case analysis)	$20,429 (complete case analysis)	SSI (multiple imputation) $2,866	Not reported
					SSI (complete case analysis): $1,825	
Pollard (2006) [36]	Sex, age, fracture type, ASA grade, pre-fracture residence type, operation, social dependency & mobility scores	Hospital stay, Antibiotics, outpatient treatment, theatre time, prosthetic costs, radiology, physiotherapy	$44,157	$13,043	$31,114	SSI: 80 days (median)
						Non-SSI: 28 days (median)
Thakar (2010) [39]	Sex, age, fracture type, ASA grade, operation, pre-fracture residence type, social dependency & mobility	Theatre time, prosthetic costs, radiology and pharmaceuticals	Superficial SSI: $30,193	Superficial SSI control: $13,987	Superficial SSI: $16,206	Superficial SSI: 62.5 days
			Deep SSI: $39,299	Deep SSI control: $13,631	Deep SSI: $25,669	Superficial SSI control: 35 days
						Deep SSI: 79.3 days
						Deep SSI matched control: 34.3 days

All costs were inflated and converted to 2017 international dollars where appropriate.

ASA grade, American Society of Anaesthesiologists; DRG, Diagnosis-related group; GP, General Practitioner; HAI, Hospital Acquired Infection; LOS, Length of stay; NNIS, Nosocomial Infection Surveillance System risk index; SSI, Surgical Site Infection

To facilitate a cost comparison across studies a specified year for which the costs are applicable allows for the findings to be inflated correctly. The cost year was not stated in six studies [35, 40, 43, 45, 46, 50]. Transparency on the amount that each cost component is contributing to the additional cost of SSI clarifies which aspects of medical care are driving the additional cost burden. However, the additional cost of SSI was not broken down into their cost components in seven studies [36, 38, 42, 45, 47, 49].

All but one study restricted costs to the perspective of the health care payer. Alfonso et al [41] widened the perspective to societal and looked at direct and indirect costs associated with SSI including hospital, primary care, informal care, and productivity loss.

#### Resource use

The reporting of resource use of SSI and non-SSI patients was inconsistent across studies. Beyond the main resource item of hospital length of stay, there was little detail on the differential resource use of SSI and non-SSI patients. Alfonso et al [41] (Spain) reported that patients with an SSI had significantly longer durations of use for hospital consumables (catheters, and antibiotics) compared with patients without an SSI. However, resource use details were omitted on general practitioner/ nurse visits and the level of informal care needed. Reilly et al [44] (UK) presented a breakdown of resource use for SSI patients only.

#### Cost of surgical site infection

Overall there was a lack of detail in the reporting of costs for SSI and non-SSI patients. Average costs of both the respective SSI and non-SSI patients groups were omitted for the majority of studies [37, 41, 42, 44–49].

Lynch et al [43] had the lowest relative magnitude of cost difference with SSI costs being 1.73 times higher than non-SSI costs. The authors had estimated the costs of SSI and non-SSI patients as $3,678 and $2,116 respectively [43].

Pollard et al [36] reported the highest relative magnitude of cost difference with SSI costs being 3.39 times higher than non-SSI costs. For elderly proximal femoral fracture surgery patients, they had estimated the costs of SSI and non-SSI patients to be $44,157 and $13,043 respectively. Their inclusion criteria meant that the SSI patients were those who specifically needed further surgery, representing an upper estimate of the additional costs of an SSI.

While all eligible studies had to present a cost difference between SSI and non-SSI patients, there was a lack of reporting of the average costs for the SSI and non-SSI patient groups used to calculate the difference (Table 2). All studies showed an elevated cost of SSI relative to non-SSI patients. The additional medical costs of SSI, which included costs incurred by the hospital and health system, ranged from $21 to $34,001 per patient.

The lowest additional cost associated of SSI was estimated in a Danish study assessing the cost-effectiveness of incisional negative pressure wound therapy in obese women after caesarean section. In addition to the cost-effectiveness results, the study also provided a per-patient cost of superficial SSI and deep SSI compared with patients who did not suffer an SSI. The superficial SSI was defined as requiring antibiotic treatment for an infection at the surgical site within the first 30 days after the caesarean section and not requiring further surgery. The highest additional health care cost associated with SSI was estimated by Weber et al [47]. While the average additional cost of all SSI patients was $17,060, an organ space SSI approximately doubled the additional cost of an SSI in their case-control designed study.

Alfonso et al [41] (Spain) was the only study to adopt a broader societal perspective and included the cost of productivity loss, informal care and health care costs. They estimated the cost associated with SSI to be an additional $145,366 per patient. This estimate comprised productivity costs (78.7%) with carer costs (10.8%) and health costs (10.5%) making up the remainder. Including only the health care costs made the additional cost of SSI $15,733 per patient.

#### Checklist

All studies were compared against a modified reporting Müller COI study checklist (see S1 Table for scores). Alfonso et al [41] achieved the highest number of items (23 points) in the checklist with detailed descriptions of the methods used to estimate the additional costs of SSI. Turtainen et al [45] achieved the lowest score (11 points) in the checklist with little to no description in the study on what was included in the SSI cost estimate and how it was derived. In general, studies scored relatively poorly in the evaluation methods and presentation of results section of the checklist but highly in the discussion and conclusions sections.

### Part 2: LMIC literature search

**Fig 2:**
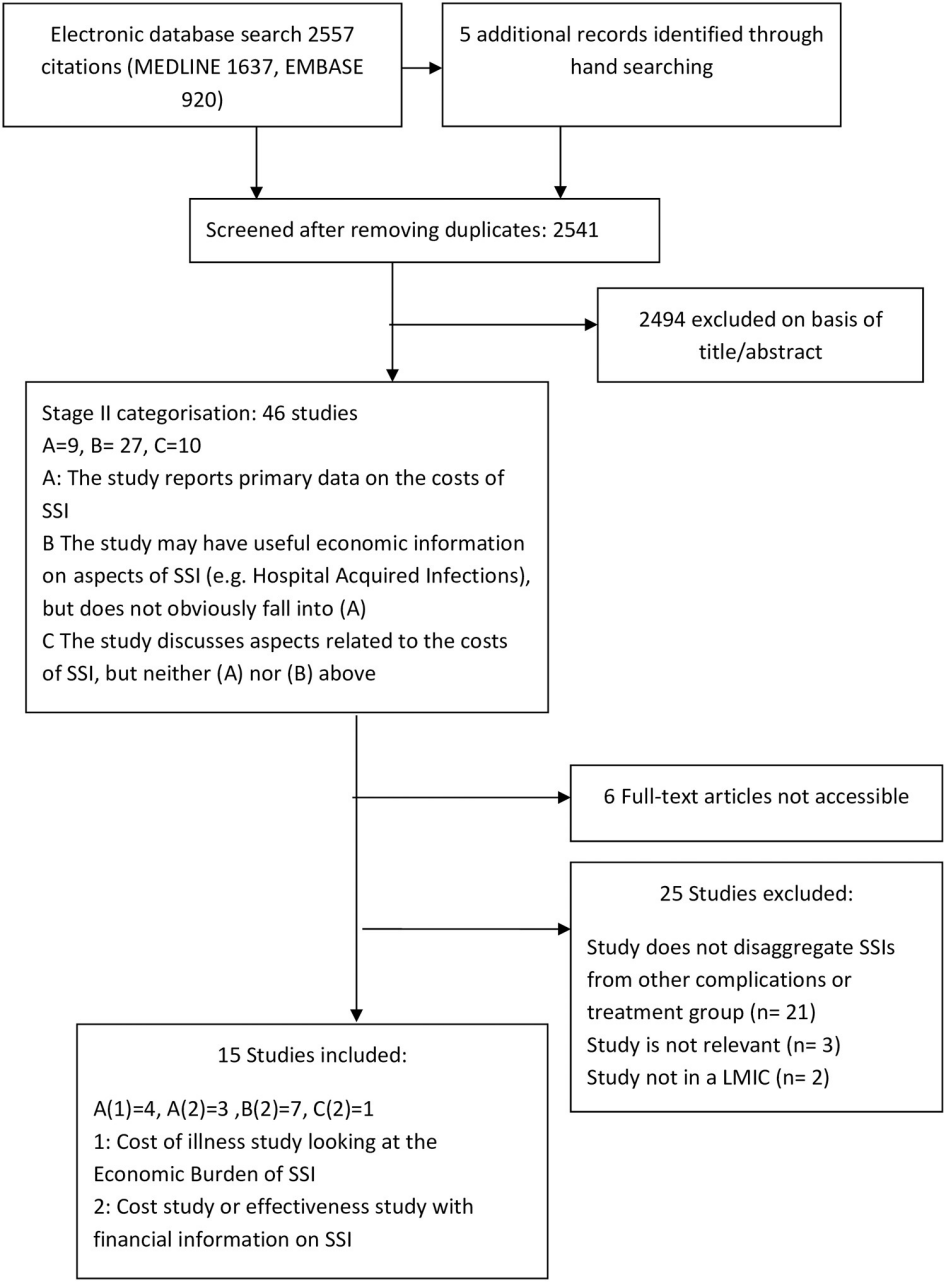
PRISMA Diagram of LMIC search.

The LMIC studies electronic database search yielded 2,557 citations. Five additional records were identified through hand searching references of included papers. Fig 2 presents a flow diagram of the selection process. Fifteen studies met the inclusion criteria.

#### General study characteristics

The fifteen studies were published from 2003 to 2018 and data collection spanned 1999 to 2015. Studies were based in Turkey (n = 3) [52–54], China (n = 2) [55, 56], Jordan (n = 2) [57, 58], Thailand (n = 2) [59, 60], Brazil (n = 1) [61], Egypt (n = 1) [62], India (n = 1) [63], Mexico (n = 1) [64],Rwanda (n = 1) [65], and South Africa (n = 1) [66]. According to the World Bank classifications, the studies were part of the following income groups: Low Income Country (n = 1) [65], Lower Middle Income (n = 2) [62, 63] and Upper Middle Income (n = 12) [52–61, 64, 66]. Table 3 shows general characteristics of each study included in the review.

**Table 3:** LMIC study characteristics.

Lead author (Year) [Income Group]	Country	Patient population	Setting	Study aim	Type of study	Number of SSI & Comparator	Period of data collection
**Cardio-thoracic surgery**							
Al-Zaru (2011) [57] [Upper Middle Income]	Jordan	CABG adult patients	Teaching hospital	Assess clinical & economic impact of SSIs	Retrospective comparative study, cost estimation	106 SSI/ 525 Non-SSI	2005–2008
Coskun (2005) [54] [Upper Middle Income]	Turkey	CABG adult patients referred back with Sternal SSI	Private hospital	Evaluate costs & outcomes for Sternal SSI	Prospective surveillance	88 SSI/88 Non-SSI	1999–2002
**General Surgery or multiple surgical categories**							
Dramowski (2016) [66] [Upper Middle Income]	South Africa	Paediatric surgery, orthopaedics and urology patients	Teaching Children hospital	Investigate burden & risk factors of HAI	Prospective surveillance	21 SSI/ 1022 Non-SSI	2014–2015
Galal (2011) [62] [Lower Middle Income]	Egypt	Surgery patients 21–60 years	Teaching hospital	Compare different sutures for SSI reduction	Prospective randomised double blind study	50 SSI/400 Non-SSI	Not reported
Porras-Hernández (2003) [64] [Upper Middle Income]	Mexico	Neurological, cardiovascular & general surgery patients, younger than 18 years	Tertiary teaching paediatric hospital	Determine the incidence of SSI	Prospective study	80 SSI / 348 Non-SSI	1998–1999
Siribumrungwong (2015) [60] [Upper Middle Income]	Thailand	Varicose Vein patients	Teaching hospital	Economic evaluation of interventions for great saphenous vein ablation	Prospective cohort study / economic analysis	4 SSI/ 73 Non-SSI	2011–2013
Tiwari (2013) [63] [Lower Middle Income]	India	Adult patients with at least 48 hours hospital stay	Private tertiary care hospital	Assess the costs associated with HAIs	Retrospective comparative study / cost analysis	4 SSI/ 104 Non-SSI	2008–2009
**Gastrointestinal surgery**							
Liu (2018) [56] [Upper Middle Income]	China	Colorectal cancer adult patients who had tumour surgically removed	Tertiary public hospital	Economic burden caused by HAIs	Retrospective surveillance / cost analysis	20 SSIs/ 38 Non-SSI	2015
Özmen (2016) [52] [Upper Middle Income]	Turkey	Elective gastric surgery cancer patients	Teaching hospital	Factors affecting SSI rate after elective gastric cancer surgery	Prospective observational cohort study	10 SSI/ 42 Non-SSI	2013
Phothong (2015) [59] [Upper Middle Income]	Thailand	Patients with sigmoid cancer	Teaching hospital	Outcomes and treatment costs following a sigmoidectomy	Retrospective review / Economic analysis	6 SSI/ 44 Non-SSI	2008–2013
Silverstein (2016) [65] [Low Income]	Rwanda	Biliary disease surgery patients	Referral military hospital, secondary and tertiary care	Laparoscopic cholecystectomy versus an open approach	Economic analysis / Cohort study	Not reported	Not reported
**Gynaecological Surgery**							
Köşüş (2009) [53] [Upper Middle Income]	Turkey	Women who had caesarean surgery	Private hospital	Trial on the prevention of post-caesarean wound infection	Randomised prospective study	38 SSI/ 76 Non-SSI	2004–2007
**Cardio & Neurological surgery**							
Zhou (2015) [55] [Upper Middle Income]	China	Patients who had a craniocerebral operation	Tertiary care hospital	Cost-benefit analysis of SSI control	Prospective study / economic analysis	12 SSI/ 588 Non-SSI	2009–2012
Hweidi (2018) [58] [Upper Middle Income]	Jordan	Adult patients who had a craniocerebral operation	Teaching hospital	Estimate the additional healthcare costs attributable to SSI	Retrospective case control study	32 SSI/ 32 Non-SSI	2009–2015
**Orthopaedic surgery**							
Dal-paz (2010) [61] [Upper Middle Income]	Brazil	Total knee arthroplasty patients	Tertiary level teaching hospital	Estimate the additional cost of nosocomial infections	Retrospective observational cohort study / cost analysis	34 SSI/ Non-SSI cases not reported	2006–2007

All costs were inflated and converted to 2017 international dollars where appropriate

CABG, Coronary Artery Bypass Graft; HAI, Hospital Acquired Infection; SSI, Surgical Site Infection;

#### Definition of SSI

The Center of Disease Control guidelines were used in the majority of the studies to define an SSI [52–57, 61–64, 66]. However, three studies lacked a definition of what constituted a SSI [59, 60, 65]. One study classified a SSI based on the wound discharge culture or other SSI suggestive signs and symptoms but these were not elaborated further [58].

Post-discharge SSIs cannot be detected where there is no follow-up. In this review, patients were not followed up after hospital discharge or it was not indicated in many of the studies [55, 56, 58–61, 63, 65, 66]. Where follow-up was specified, it was only recorded if the patient happened to return to the index hospital in two studies [54, 57]. The only specified method of follow-up in the studies was attendance of an outpatient clinic attendance a month after the patient’s operation [52, 54, 62, 64].

#### Patient matching

When estimating the additional cost burden of SSI, most of the studies did not make any adjustments in the comparison with non-SSI patients or it was unclear if adjustment had been used (Table 3). Justification on the inclusion of the patient matching variables was only given in one of the six studies where patient matching was utilised [56].

#### Setting & procedure

The setting where the findings are derived from were mainly public teaching hospitals [41, 52, 57–62, 64, 66] with three based in private hospitals [53–55, 63]. All the studies were based in single centres. Surgical procedures ranged from general surgery or multiple surgical categories (n = 5), oncological procedures (n = 4), cardiothoracic (n = 2), orthopaedic (n = 1), gastric (n = 1), general, cardiac and neurosurgery (n = 2). The patient population was broader in the LMIC studies and varied from children (n = 2), adults (n = 12) and pregnant women (n = 1). For the studies with SSI patients taken from multiple surgical categories, none reported costs of SSI by surgical category.

SSI severity increased the additional cost of SSI [54, 56]. A subgroup analysis of one study had low sample sizes for the superficial (n = 13), subcutaneous (n = 6) and deep soft SSIs (n = 1)^53^. Another study compared the severity of infections in three different types of surgical procedure, however, the reported cost was for all cases [64].

In general, studies tended to have a low number of SSI patients with the sample size of SSI patients in each study ranging from 4 patients [60, 63] to 106 patients [57]. Six studies had twenty or fewer SSI patients [52, 53, 56, 59, 60, 63].

#### Cost components

All studies estimated direct medical costs (Table 4). The lack of follow-up of patients beyond discharge limited most of the studies to report only inpatient hospital costs. One study had attempted to measure the direct non-medical costs, however the authors did not report it as a cost of an SSI [65]. Most studies did not report the relevant year for the cost estimation (S2 Table). The majority of studies did not break down the extent to which each cost component makes up the costs of SSI and non-SSI patients. Where cost components were reported in studies, it either included both SSI and non-SSI patients [55, 59, 65] or was limited to only SSI patients [53, 54, 61].

**Table 4:** Costs of SSI in LMIC studies.

Lead author (Year)	Adjusted group comparison	Costs included	Average cost SSI patients	Average costs Non-SSI patients	Additional cost of SSI	Length of Stay
**Cardio-thoracic surgery**						
Al-Zaru (2011) [57]	Unadjusted comparison	Hospital stay, medications, radiology, microbiological & lab tests	$31,666	$22,329	$9,337	SSI: 16.7 days Non-SSI: 7.8 days
Coskun (2005) [54]	Age & sex	Medication, examination and lab test, hospital stay, additional operation	Not reported	Not reported	Deep: $23,408 Superficial: $12,782	Deep SSI: Extra 35 days Superficial SSI: Extra 21 days
**General surgery**						
Dramowski (2016) [68]	Age, ward, preoperative length of stay	Hospital length of stay, laboratory investigations, radiology and pharmacy cost	Not reported	Not reported	$1,546	SSI median excess days: 4 days Non-SSI: not reported
Galal (2011) [62]	Unadjusted comparison	Hospital stay	$2,465	$610	$1,855	SSI: 7.10 days Non-SSI: 3.39 days
Porras-Hernández (2003) [64]	Unadjusted comparison	Hospital stay (excluding antibiotics)	Not reported	Not reported	$2,164	SSI: 13 days Non-SSI: 6 days
Siribumrungwong (2015) [60]	Not reported	Unspecified hospital costs	Not reported	Not reported	$174	Not reported
Tiwari (2013) [63]	Matched groups of HAI and non-HAI by age, diagnosis, illness severity	Consumables, hospital room, medications, investigations, blood components, consultation	$37,295	$7,685	$29,610	SSI: Not reported Non-SSI: 9 days
**Gastrointestinal surgery**						
Liu (2018) [58]	Age, sex, comorbidity, disease, and prior surgeries	Medication, equipment & supplies, diagnostics	Not reported	$11,691	Overall: $1,410 Superficial: $462 Subcutaneous SSI: $2,386 Deep soft SSI: $17,094	SSI: Not reported Non-SSI: 22 days (median)
Özmen (2016) [52]	Unadjusted comparison	Hospital stay	$4,195	$4,872	SSI patients had lower costs	SSI: 5.27 days Non-SSI: 5.40 days
Phothong (2015) [59]	Unadjusted comparison	Room charges, theatre time, medication, anaesthesia, equipment & laboratory charges & nursing	$12,109	$5,960	$6,149	SSI: 23.5 days Non-SSI: 9.8 days
Silverstein (2016) [65]	Not reported	Unclear	Not reported	Not reported	$483	Not reported
**Cardio & Neurological surgery**						
Zhou (2015) [55]	Age, sex, operation type, incision type, operation date, & physical status	Medication, equipment, lab test, treatment, exams and additional surgeries	$16,979	$10,240	$6,739	SSI: 29 days Non-SSI: 17.25 days
Hweidi (2018) [58]	Age, sex, index diagnosis, admission month	Length of stay, antibiotics, reoperation	$34,872	$15,974	$18,899	SSI: 30.15 days Non-SSI: 6.98 days
**Gynaecological surgery**						
Köşüş (2009) [53]	Unadjusted comparison	Preventative antibiotics, hospital readmission and out-patient	$1,736	$0	$1,736	Two SSI patients had 7 days readmission. None for Non-SSI patients
**Orthopaedic surgery**						
Dal-paz (2010) [61]	Unadjusted comparison	Hospital stay, lab and imagining test, additional operations and antibiotics	Not reported	Not reported	$3,865	SSI: Extra 29.7 days Non-SSI not reported

All costs were inflated and converted to 2017 international dollars where appropriate.

CABG, Coronary Artery Bypass Graft; HAI, Hospital Acquired Infection; SSI, Surgical Site Infection;

#### Resource use

There was no reporting of resource use of SSI and non-SSI patients beyond hospital length of stay in any of the studies. There was partial reporting on the additional procedures or investigations for SSI [54, 61] but no detail on the total resource use by SSI and non-SSI patients.

#### Cost of surgical site infection

The additional cost of SSI varied considerably across the studies. All but one study showed an elevated cost of SSI relative to non-SSI patients. The study by Özmen et al [52] (Turkey) study looked at outcomes of patients after elective gastric cancer surgery and found that the unspecified hospital costs were non-significantly lower for superficial SSI patients compared to non-SSI patients. The calculations behind the lower SSI cost was unclear given that overall hospital costs were higher than either of the mean costs of the patient groups (SSI and non-SSI).

The additional cost of SSI ranged from $174 (Thailand) [60] to $29,610 (India) [63]. The lowest additional cost of SSI was from a study by Siribumrungwong et al [60]. Their SSI cost was made up of undefined hospital costs of four SSI patients with no detail of the non-SSI comparator group. The highest additional cost of SSI was from a study by Tiwari et al [63]. For their four patients who suffered an SSI, drug acquisition costs, length of stay and antimicrobial drugs were the main cost drivers.

Clarity on the relative magnitude of difference in cost between SSI and non-SSI patients was mixed. Half of studies did not present average costs of both SSI and non-SSI patients. The lowest relative magnitude of difference in reported costs was in Jordan where SSI costs were 1.4 times higher than non-SSI costs [59]. However, it is unclear what cost items are the major contributors of the additional costs. They had estimated the costs of SSI and non-SSI patients as $31,666 and $22,329, respectively. The highest relative magnitude of difference in costs was in India where Tiwari et al [63] found that SSI costs were 4.8 times higher than non-SSI costs [63]. The authors had estimated the costs of SSI and non-SSI patients as $37,295 and $7,685 respectively.

#### Checklist

For the COI checklist, the studies achieved on average a score of 11.07 out a maximum of possible score of 36. The lack of a stated perspective and cost year reduced the scores of many of the LMIC studies. Dramowski et al [66] scored the highest number of items (16) in the discussion and conclusion. The lack of description of pertinent study items meant that Porras-Hernández et al [66] scored the lowest (6).

### Part 3: Comparison between HIC European countries and LMICs

The CDC criteria were used for SSI diagnosis by most studies in both settings. The biggest methodological difference between the HIC and LMIC settings was the use of adjusted analyses for comparing SSI and non-SSI patients. Most European studies used patient matching while the opposite was true for LMIC studies. Multicentre study settings were only present in the European studies. Slightly more European studies had follow up beyond discharge but the follow-up methods varied. Sample sizes of SSI patients tended to be higher in the European studies. On the other hand, LMIC studies had marginally better reporting of the average costs of the SSI and non-SSI patient groups.

For the COI reporting checklist, the European studies achieved a higher score on average compared with the LMIC studies. In both settings, studies tended to score highly in the discussion and conclusion checklist but poorly on the evaluation methods and result presentation sections.

#### Statement of principal findings

This review assessed the estimated the cost burden of SSIs in the reported literature for both LMICs and a selection of European High Income Countries.

For medical costs, the additional cost of SSI was $21 to $34,000 in European studies while the additional cost attributed to SSI ranged from $174 to $29,610 in LMICs. The huge range of costs in both settings reflects the difficulty associated with accurately estimating the costs attributable to SSI and consequently limited cross-study comparability of findings. Five main challenges to the estimation of the costs are summarised below:

1. Time horizon for capturing an SSI
2. Choice of comparator
3. Over reliance on single centre studies and small number of patients with SSI
4. Under representation of Low Income and Lower Middle Income Countries in the literature
5. Inconsistency in consideration of costs and narrow cost perspective.

1. ***Time horizon and follow-up*:** Studies from both settings used the CDC criteria to define SSI, but the lack of follow-up in LMIC studies failed to meet the recommended time needed to detect an SSI. According to the CDC, the specified time horizon for an SSI to occur is up to 30 days post-surgery for non-implant operations and up to 12 months for implant operations. Where no follow-up exists, there is a risk of underestimating the true number of SSI patients and skewing the cost burden information to only patients with an inpatient SSI. The type of follow-up method will affect the detection rate but this was rarely mentioned in studies. Inadequate IT infrastructure in LMIC healthcare systems has been implicated as the cause of poor follow up through health care pathways [67].
2. ***The choice of comparator*** was important in the estimating the additional cost burden of SSI. Most LMIC studies did not use any adjustments for potential confounders which risked producing a false estimation (overestimate or underestimate) of SSI costs due to an imbalance in the characteristics of the comparators. For example, some of the differences in costs between SSI patients and non-SSI patients could be due to greater levels of comorbidity in one group, causing a higher estimated additional cost for SSI than may otherwise be true. In contrast, the majority of the European studies did make adjustments for potential confounders but few gave justification for the included matching variables. Proper consideration of matching variables can help avoid the problem of undermatching or overmatching in case-control studies [25].
3. **Over reliance on single centre studies:** Both settings had an overreliance on single centre studies and the lack of multi-centre settings affected the representativeness of the findings. Greater numbers of hospitals participating and more diversity in hospital settings for each study would help strengthen the applicability and robustness of any findings. Some studies with a patient population across multiple surgical categories indicated differential additional SSI costs by procedure. However, this was only reported in the European studies and there was no clear signal on which procedures would be the costliest across these studies. Some studies had low numbers of SSI patients; cost estimation with a small sample size are prone to unreliability and imprecision. This has an impact on the interpretation of the results given that the differences in costs between SSI patients and non-SSI patients could be driven by chance or extreme values. In general, the European studies had more patients, but this could be as a result of better SSI surveillance.
4. **Lack of studies in Low Income and Lower Middle Income countries:** The LMIC studies found in the review span across different continents, patient populations, surgical procedures, income levels, health systems and cultures. However, there was an underrepresentation of studies in Low Income countries and Lower Middle Income countries making the generalisability of the overall findings to these settings more difficult.
5. **Inconsistency in consideration of costs and narrow cost perspective:** The type of costs included will have a direct impact on the estimation of SSI costs. The cross-country cost comparison of SSI was hindered by the absence of a standardized approach in the basket of cost items included. However, even when a standardized approach is adopted as in a multinational randomised control trial, costs and resource use will differ across countries [68]. Variations in clinical practice and relative prices across countries will affect the transferability of healthcare resource use and costs [69]. Despite an SSI has far-reaching resource use implications for the healthcare system, patient and community, costs from the patient’s perspective were not considered in any LMIC study. The absence of patient and societal costs are concerning given the relatively high out of pocket expenditure faced by patients in LMICs. Lack of consideration of these costs is likely to underestimate the true cost burden of SSI, and one of the European studies found that the addition of informal care alone doubled the costs associated with SSI [41].

#### Strengths and weaknesses

The strength of this study is that it is the first systematic review to specifically investigate the economic impact of SSI in LMICs. By including a parallel review of SSI with HICs in Europe, the review offers new insight into the methodological considerations and the potential data gaps in SSI cost studies from the contrasting settings.

A limitation relates to the use of an *implied* PPP exchange rate for some of the LMIC settings and the English language restriction for the article inclusion criteria. A PPP exchange rate is used to adjust for the cost of living differences between countries. Relying on implied PPP rates for adjusting the comparative cost results is likely to introduce measurement error in the study findings compared to those using official PPP rates [70]. A previous study looking at risk factors for child conduct problems and youth violence in LMICs reported that including only English language studies was likely to have reduced the number of potentially relevant articles by around 15% [71].

#### Comparison with other studies

Previous systematic reviews have looked at the costs of a SSI, mainly in high income countries [5, 72, 73]. Similar issues were encountered on the lack of standardized approach, insufficient detail on how costs were derived, and the failure to include societal costs. To better articulate the first two study issues, the present review added the use of a cost of illness reporting checklist to give an indication of the study transparency and comparability. In contrast to the previous systematic reviews, the search criteria of the present study were not limited by date to be as inclusive as possible. A previous systematic review established that many essential surgical interventions are cost-effective in resource poor countries [74]. However, complications such as SSI can impose unforeseen additional costs in these countries, which are overlooked by most of the studies included in the paper.

#### Implications for practice

An SSI is the most common hospital acquired infection in LMICs [75]. Preventing SSIs will decrease the financial burden of both the patient and health system. Hospital bed overcrowding is problematic in LMICs [76–78] and any reduction in SSIs would help to increase capacity in bed days.

There is need for multicentre studies with large number of SSI patients to capture relevant costs and consequences of the infection across settings. The use of a standardized data collection pathway will help improve cross-study comparability. Future studies should include more detailed information on analytic approaches in the methods along with rationale and discussion of their likely impact on results. Ideally, reporting should include resource use, costs and cost categories of SSI and non-SSI patients to give more context on the key influences for the cost difference between patient groups. The identification, measurement and collection of costs should as far as possible take a societal perspective to appropriately encompass all healthcare, patient and wider society costs that may be affected by an SSI. The costs of inpatient SSI and outpatient SSI need to be differentiated given that the former is plausibly more expensive from increased inpatient bed days. Subgroup analysis would allow the heterogeneity to be examined between these groups instead of being masked in overall figures.

## Conclusions

An SSI represents a financial burden in both high income and LMICs settings. The magnitude of the cost difference depends on the SSI definition used, severity of SSI, patient population, choice of comparator, hospital setting, and cost items included. Huge heterogeneity in design and lack of transparency has made it difficult to draw meaningful comparison across studies and countries.

We suggest that future studies endeavour to achieve the most appropriate time horizon to include appropriate complications, focus on a comparator that has a degree of matching of patient characteristics, and researchers should limit their focus on single centre studies to increase generalisability. These three items are typically within the gift of researchers during the design stage. The impact of SSI in low-income countries is likely to be severe and more research in these setting is required with particular care on choosing the right perspective for the collection of cost data, which is key to ensuring the appropriate financial burden captured. Agreement on what would the composition of a standardised basket of items of costs to include would also be extremely helpful.

## Supporting information

S1 Table Cost information included in each European study. (DOCX)

S2 Table Cost information included in each LMIC study. (DOCX)

S1 File LMIC search strategy medline. (DOCX)

S2 File LMIC search strategy embase. (DOCX)

S3 File Europe search strategy medline. (DOCX)

S4 File Europe search strategy embase. (DOCX)

S1 Checklist PRISMA 2009 checklist. (DOC)
