# Supplementary material for: Surgical site infection and costs in low- and middle-income countries: A systematic review of the economic burden
Source: PLoS One. 2020 Jun 4;15(6):e0232960. doi: 10.1371/journal.pone.0232960 (PMC7272045; doi:10.1371/journal.pone.0232960)
Supplement: S1 Table — (DOCX) [file pone.0232960.s001.docx]

## S1 Table. Cost information included in each European study

| Study | Alfonso | Defez | Edwards | Graf | Lynch | Jenks | Hyldig | Parker | Pollard | Reilly | Tanner | Thakar | Turtiainen | Vegas | Vrijens | Weber |
| --- | --- | --- | --- | --- | --- | --- | --- | --- | --- | --- | --- | --- | --- | --- | --- | --- |
| Cost year | Y | NR | N | NR | Y | NR | Y | Y | Y | Y | Y | Y | NR | Y | Y | Y |
| Cost subdivision | Y | Y | Y | Y | Y | N | N | Y | N | N | Y | N | N | NA | NA | N |
| Hospital stay | Y | Y | Y | Y | Y | Y | Y | Y | Y | Y | Y | Y | Y | Y | Y | Y |
| Diagnostics | Y | Y | Y | Y | N | Y | Y | Y | N | N | Y | Y | N | N | N | N |
| Medication (no Antibiotics) | N | N | N | N | Y | N | Y | Y | N | N | N | Y | N | N | N | Y |
| Antibiotics | Y | Y | Y | N | Y | N | Y | Y | Y | N | N | Y | N | N | N | Y |
| Readmission | Y | N | N | N | N | Y | Y | Y | N | Y | Y | N | N | N | N | N |
| Outpatient | Y | N | N | N | Y | N | Y | Y | Y | Y | Y | N | Y | N | N | N |
| Patient or Family costs | Y | N | N | N | Y | N | N | N | N | Y | N | N | N | N | N | N |
| COI reporting checklist score | 21 | 15 | 13 | 12 | 19 | 18 | 18 | 20 | 10 | 14 | 15 | 14 | 11 | 13 | 17 | 16 |
