# Supplementary material for: Surgical site infection and costs in low- and middle-income countries: A systematic review of the economic burden
Source: PLoS One. 2020 Jun 4;15(6):e0232960. doi: 10.1371/journal.pone.0232960 (PMC7272045; doi:10.1371/journal.pone.0232960)
Supplement: S2 Table — (DOCX) [file pone.0232960.s002.docx]

## S2 Table. Cost information included in each LMIC study

| **Study** | Al-Zaru | Coskun | Dal-paz | Dramowski | Galal | Hweidi | Köşüş | Liu | Özmen | Phothong | Porras-Hernández | Silverstein | Siribumrun-gwong | Tiwari | Zhou |
| --- | --- | --- | --- | --- | --- | --- | --- | --- | --- | --- | --- | --- | --- | --- | --- |
| Cost year | NR | NR | Y | Y | NR | NR | NR | NR | NR | NR | NR | Y | N | NR | N |
| Cost subdivision | N | Y | Y | N | NA | N | N | N | N | Y | NA | N | N | Y | N |
| Hospital stay | Y | Y | Y | Y | Y | Y | Y | Y | UC | Y | Y | N | N | Y | Y |
| Diagnostics | Y | Y | Y | Y | N | N | N | Y | UC | Y | N | N | N | Y | Y |
| Medication | N | N | N | UC | N | N | N | Y | UC | Y | N | N | N | Y | Y |
| Antibiotics | Y | Y | Y | Y | N | Y | Y | N | UC | Y | N | N | N | Y | Y |
| Readmission for surgery | N | N | Y | N | N | Y | N | N | N | N | N | N | N | N | N |
| Outpatient | N | N | N | N | N | N | Y | N | N | N | N | N | N | N | N |
| Patient or Family | N | N | N | N | N | N | N | N | N | N | N | N | N | N | N |
| COI reporting checklist score | 10 | 11 | 11 | 16 | 9 | 13 | 11 | 14 | 7 | 11 | 6 | 12 | 9 | 12 | 17 |
| Y= Yes; N= No; NA= Not applicable; UC= Unclear | | | | | | | | | | | | | | | |
