# Supplementary material for: Surgical site infection and costs in low- and middle-income countries: A systematic review of the economic burden
Source: PLoS One. 2020 Jun 4;15(6):e0232960. doi: 10.1371/journal.pone.0232960 (PMC7272045; doi:10.1371/journal.pone.0232960)
Supplement: S3 File — (DOCX) [file pone.0232960.s005.docx]

## S3 File. Europe Search strategy Medline

1. economics/
2. cost of illness/
3. exp health care costs/
4. economic value of life/
5. exp economics medical/
6. exp economics hospital/
7. economics pharmaceutical/
8. exp fees/ and charges/
9. (econom$ or cost or costs or costly or costing or price or pricing or pharmacoeconomic$).tw.
10. (expenditure$ not energy).tw.
11. (value adj1 money).tw.
12. budget$.tw.
13. 1 or 2 or 3 or 4 or 5 or 6 or 7 or 8 or 9 or 10 or 11 or 12
14. (wound infection adj8 surgery).tw.
15. Wound infec$.tw.
16. exp Wound Infection/
17. Surgical wound infection.tw.
18. exp Surgical Wound Infection/
19. 14 or 15 or 16 or 17 or 18
20. 13 and 19
21. exp United Kingdom/ or exp iceland/ or exp switzerland/ or exp Austria/ or exp Wales/ or exp scotland/ or exp Northern Ireland/ or exp Norway/ or exp Belgium/ or exp sweden/ or exp Czech Republic/ or exp Estonia/ or exp Greece/ or exp Spain/ or exp England/ or exp France/ or exp Finland/ or exp Germany/ or exp Denmark/ or exp Italy/ or exp Hungary/ or exp Ireland/ or exp Latvia/ or exp lithuania/ or exp luxembourg/ or exp netherlands/ or exp poland/ or exp portugal/ or exp slovakia/ or exp slovenia/
22. 20 and 21
