# Supplementary material for: Surgical site infection and costs in low- and middle-income countries: A systematic review of the economic burden
Source: PLoS One. 2020 Jun 4;15(6):e0232960. doi: 10.1371/journal.pone.0232960 (PMC7272045; doi:10.1371/journal.pone.0232960)
Supplement: S4 File — (DOCX) [file pone.0232960.s006.docx]

## S4 File. Europe Search strategy Embase

1. cost benefit analysis/
2. cost effectiveness analysis/
3. cost minimization analysis/
4. cost utility analysis/
5. economic evaluation/
6. (cost or costs or costed or costly or costing).tw.
7. (economic$ or pharmacoeconomic$ or price$ or pricing).tw.
8. or/1-7
9. exp Surgical Wound Infection/
10. exp Wound Infection/
11. exp Surgical wound/
12. exp Surgical infection/
13. Surgical wound infection.tw.
14. (wound infection adj8 surgery).tw.
15. Wound infec$.tw.
16. or/9-15
17. and/8,16
18. exp United Kingdom/ or exp iceland/ or exp switzerland/ or exp Austria/ or exp Wales/ or exp scotland/ or exp Northern Ireland/ or exp Norway/ or exp Belgium/ or exp sweden/ or exp Czech Republic/ or exp Estonia/ or exp Greece/ or exp Spain/ or exp England/ or exp France/ or exp Finland/ or exp Germany/ or exp Denmark/ or exp Italy/ or exp Hungary/ or exp Ireland/ or exp Latvia/ or exp lithuania/ or exp luxembourg/ or exp netherlands/ or exp poland/ or exp portugal/ or exp slovakia/ or exp slovenia/
19. and/17-18
